# Optimization of TripleTOF spectral simulation and library searching for confident localization of phosphorylation sites

**DOI:** 10.1371/journal.pone.0225885

**Published:** 2019-12-02

**Authors:** Ayano Takai, Tomoya Tsubosaka, Yasuhiro Hirano, Naoki Hayakawa, Fumitaka Tani, Pekka Haapaniemi, Veronika Suni, Susumu Y. Imanishi

**Affiliations:** 1 Faculty of Pharmacy, Meijo University, Nagoya, Japan; 2 Turku Bioscience Centre, University of Turku and Åbo Akademi University, Turku, Finland; 3 Turku Centre for Computer Science, Turku, Finland; Swiss Institute of Bioinformatics, SWITZERLAND

## Abstract

Tandem mass spectrometry (MS/MS) has been used in analysis of proteins and their post-translational modifications. A recently developed data analysis method, which simulates MS/MS spectra of phosphopeptides and performs spectral library searching using SpectraST, facilitates confident localization of phosphorylation sites. However, its performance has been evaluated only on MS/MS spectra acquired using Orbitrap HCD mass spectrometers so far. In this study, we have investigated whether this approach would be applicable to another type of mass spectrometers, and optimized the simulation and search conditions to achieve sensitive and confident site localization. Synthetic phosphopeptides and enriched K562 cell phosphopeptides were analyzed using a TripleTOF 6600 mass spectrometer before and after enzymatic dephosphorylation. Dephosphorylated peptides identified by X!Tandem database searching were subjected to spectral simulation of all possible single phosphorylations using SimPhospho software. Phosphopeptides were identified and localized by SpectraST searching against a library of the simulated spectra. Although no synthetic phosphopeptide was localized at 1% false localization rate under the previous conditions, optimization of the spectral simulation and search conditions for the TripleTOF datasets achieved the localization and improved the sensitivity. Furthermore, the optimized conditions enabled sensitive localization of K562 phosphopeptides at 1% false discovery and localization rates. These results suggest that accurate phosphopeptide simulation of TripleTOF MS/MS spectra is possible and the simulated spectral libraries can be used in SpectraST searching for confident localization of phosphorylation sites.

## Introduction

Tandem mass spectrometry (MS/MS) and sequence database searching have widely been used in the field of proteomics [1–3]. The recent advances in those techniques, and also in sample preparation, have enabled identification and quantification of thousands of phosphopeptides [4–8]. Furthermore, assisting tools such as Ascore and phosphoRS have been developed for confident localization of phosphorylation sites [9–13]. Since reversible protein phosphorylation at specific serine, threonine, tyrosine residues is a key determinant in many cellular functions [14–22], many phosphoproteomics studies have been conducted [4–8, 23–25].

In addition to database searching, spectral library searching has been available for peptide identification [26–29]. Since spectral library searching matches MS/MS spectra of peptides to the ones previously identified, it takes advantage of comparison of specific spectral features including peak intensities and neutral losses from fragments. Furthermore, open modification searches offer identifications of various post-translational modifications on library spectra [30–33]. Spectral library searching has been proven to be effective also in identification of phosphopeptides [34–36]. Hu et al. has reported computational simulation of known phosphorylation sites onto nonphosphorylated peptides, which was performed by a simple +80 Da shift of fragment ions generated by ion trap collision-induced dissociation (CID) [37, 38]. Searching by SpectraST software [28] against their spectral library supplemented with the simulated spectra was highly sensitive for phosphopeptide identifications compared to database searching [38]. However, accuracy of phosphorylation site localization by this approach was uncertain, as many false localizations appeared in our evaluation [39].

**Fig 1:**
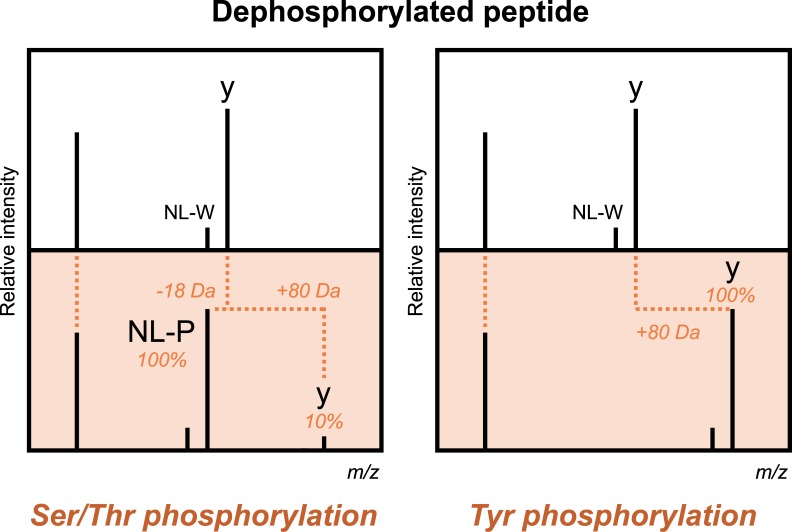
Simulation of single phosphorylation on beam-type CID spectra. From nonphosphorylated fragment ions observed in beam-type CID spectra of enzymatically dephosphorylated peptides, such as a, b, and y-series ions and their ammonia and water neutral loss ions (NL-A and -W), SimPhospho predicts serine/threonine phosphorylated ions (pS/pT intact ions) and their phosphoric acid neutral loss ions (NL-P ions) by a +80 Da shift and a -18 Da shift, respectively. Note, fragment ions with the combination of NL-A and NL-W were not taken into consideration. Tyrosine phosphorylated ions (pY intact ions) are predicted from the nonphosphorylated ions only by a +80 Da shift. In our previous study [39], intensities of pS/pT intact ions, NL-P ions, and pY intact ions relative to the nonphosphorylated ions were set to 10%, 100%, and 100%, respectively.

Recently, we have developed a phosphorylation site localization method using spectral library searching. It was demonstrated that, although a prominent neutral loss of phosphoric acid from phosphorylated serine/threonine residues readily occurs, fragmentation patterns of serine/threonine/tyrosine phosphorylated peptides and their dephosphorylated counterparts are similar in beam-type CID spectra acquired in a quadrupole time-of-flight (Q-TOF) instrument [40]. Based on this, a software tool SimPhospho was developed for beam-type CID spectra acquired in Orbitrap higher energy collisional dissociation (HCD) instruments, which enables spectral simulation of all possible single phosphorylations by predicting the diagnostic fragmentation events onto enzymatically dephosphorylated peptides (Fig 1) [39]. SpectraST searching against a spectral library of the simulated phosphopeptides achieved sensitive detection and confident localization of phosphorylation sites, even if the library was merged with publicly available large libraries. Furthermore, this simulated spectral library searching could be used to supplement database search results prior to label-free quantification [23]. Very recently, an improved version of SimPhospho has been developed with a user interface [41].

**Fig 2:**
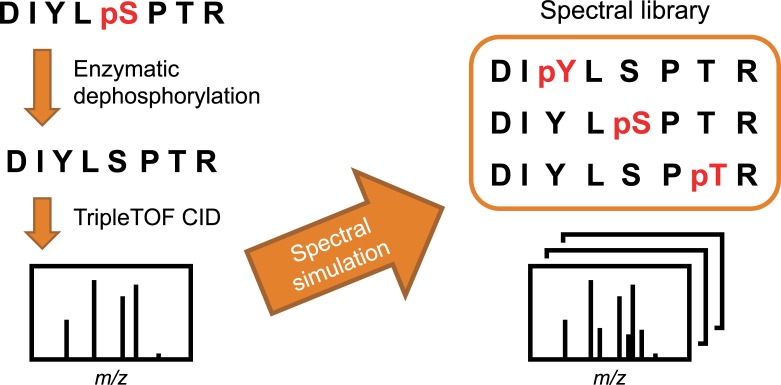
Experimental scheme of phosphopeptide simulation on TripleTOF CID spectra. SimPhospho enables spectral simulation of all possible single phosphorylations. A spectral library of simulated phosphopeptides can be used in SpectraST searching. pS: phosphoserine, pT: phosphothreonine, and pY: phosphotyrosine.

Here, we have investigated the applicability of simulated spectral library searching to beam-type CID spectra obtained using a TripleTOF 6600 Q-TOF mass spectrometer (refer to Fig 2). TripleTOF mass spectrometers are capable of high-speed MS/MS (maximum 100 spectra/second), which allows comprehensive and specific peptide quantification by data-independent acquisition, such as SWATH-MS [42]. However, also in quantitative phosphoproteomics studies, confident and sensitive identification of phosphopeptides is required prior to quantification. In this study, we have acquired TripleTOF CID spectra of synthetic phosphopeptides and K562 cell tryptic phosphopeptides before and after enzymatic dephosphorylation, and performed spectral simulation and searching for those data using SimPhospho and SpectraST software. However, this approach developed with Orbitrap HCD spectra was not readily applicable to TripleTOF CID spectra even though both fragmentation patters appear to be similar. Therefore, we have evaluated different scoring to analyze TripleTOF CID spectra and optimized simulation and search conditions. The performance of the optimized searching was evaluated in comparison to MaxQuant searching [43].

## Materials and methods

### Sample preparation

Human phosphopeptide samples were prepared according to Kauko et al. [23] with some modifications. Briefly, K562 cell lysate (1 mg protein) obtained from Promega was mixed with a phosphoprotein standard bovine α-casein (10 μg, Sigma-Aldrich), reduced with dithiothreitol, alkylated with iodoacetamide, and then digested with trypsin. After acidification with trifluoroacetic acid, aliquots of the digest (20 of 1000 μL) were desalted with a microcolumn packed with Empore C18 disk (3M) [44], for checking the digestion. The remaining digests (980 μL) were desalted with an Empore C18-SD 10mm/6mL cartridge (3M), followed by phosphopeptide enrichment with a microcolumn packed with Sachtopore-NP TiO_2_ beads (20μm, 300Å; ZirChrom) [40]. The enriched phosphopeptides were immediately desalted with the C18 microcolumn. Half of the phosphopeptide sample was subjected to enzymatic dephosphorylation with calf intestinal alkaline phosphatase (Roche), followed by desalting with the C18 microcolumn. Both the samples before and after the dephosphorylation were dissolved with 20 μL of 0.1% formic acid.

Synthetic phosphopeptides (PEPotec) containing 62 singly phosphorylated peptides (24 sequences) were obtained from Thermo Fisher Scientific. These peptides were mixed into 3 groups, where the peptide concentration was 200 pmol each based on the manufacturer information and phosphopeptide isoforms (different phosphorylation sites on the same peptide sequences) were separated from each other (see S1 Table). The peptide mixtures were desalted before and after enzymatic dephosphorylation as described above. Both the mixtures were dissolved with 100 μL of 0.1% formic acid.

### Mass spectrometry

LC-MS/MS analysis for the K562 peptides (5 μL injection) was performed in duplicates, using an Eksigent Ekspert 425 cHiPLC nanoflow LC instrument coupled to a TripleTOF 6600 Q-TOF mass spectrometer (Sciex). A 200 μm x 0.5 mm trap column and a 75 μm x 15 cm analytical column (ChromXP C18-CL, 3 μm, 120Å; Sciex) were used. The mobile phases were 0.1% formic acid in water (A) and 0.1% formic acid in acetonitrile (B). A flow rate was 300 nL/min. LC gradient elution condition was initially 5% B to 30% B (90 min) and 80% B (95–105 min). Data dependent acquisition was performed in positive ion mode. MS spectra were acquired from *m/z* 400 to *m/z* 1250 with an accumulation time of 0.1 s. The 10 most abundant ions of which charge states were from 2+ to 4+ were selected for subsequent fragmentation with rolling collision energy (0.0625 x *m/z*—X (V); X = 3 (2+), 5 (3+), or 6 (4+)). MS/MS spectra were acquired from *m/z* 100 to *m/z* 1600 with an accumulation time of 0.1 s in high sensitivity mode. Exclusion time was 20 s.

LC-MS/MS analysis for the synthetic peptides was performed in triplicates as described above, but LC gradient elution condition was initially 5% B to 40% B (10 min) and 80% B (12–20 min). The accumulation time for MS/MS and the exclusion time were modified into 0.2 s and 0 s, respectively.

### X!Tandem database search

TripleTOF data (wiff and wiffscan files) of the dephosphorylated peptides (K562 and synthetic peptides) were converted into mzXML files with Trans-Proteomic Pipeline (TPP) [45, 46] version 4.8.0. These files were subjected to database searching with X!Tandem [47] included in TPP, against a concatenated forward-reverse SwissProt database (2010_03 release, *Homo sapiens*, total 40530 sequences) used in the previous study [39]. The following search condition was used: carbamidomethylation (C) as a fixed modification, and oxidation (M), phosphorylation (S, T, and Y), acetylation (protein N-terminus), and pyrolidone (E, Q, and carbamidomethyl C) as variable modifications. Trypsin was specified as an enzyme and two missed cleavage sites were allowed. Precursor and fragment ion mass tolerances were set to 50 ppm and 0.05 Da, respectively. Probability values and false discovery rates (FDRs) were estimated using the target-decoy strategy by PeptideProphet [48] included in TPP. The searches were also tested against a SwissProt database (2018_02 release, *Homo sapiens*) supplemented with common contaminants (forward-reverse, total 40762 sequences).

### Spectral library creation

The X!Tandem search results (pepXML files) and the mzXML files of dephosphorylated peptides were subjected to spectral simulation with SimPhospho version 1 [41], which generates new pepXML and mzXML files containing only the phosphopeptides simulated from nonphosphorylated peptides. Consensus spectral libraries were built from the generated files and supplemented with decoy entries using SpectraST [28, 49, 50] version 5.0 included in TPP. For building these libraries, the nonphosphorylated peptides with a minimum probability of 0.95 were taken as the simulation templates. Different simulation conditions were used on SimPhospho and the obtained SpectraST search results were compared (see details in the Result section).

### SpectraST spectral library search

TripleTOF data of the K562 and synthetic phosphopeptides were converted into mzXML files with TPP and searched against the corresponding simulated spectral libraries with SpectraST. The following search condition was used: version 4 scoring (based on dot products of square-root intensities), precursor ion mass tolerance 3 *m/z*, and fragment ion bins 20 per *m/z*; or version 5 scoring (rank-based similarity scoring), precursor ion mass tolerance 3 *m/z*, and fragment ion mass tolerance 0.05 *m/z*. An FDR of 1% for the K562 phosphopeptides was estimated by PeptideProphet as a cutoff; however, no FDR cutoff was applied for the synthetic phosphopeptides because of the known peptide sequences and data simplicity. To estimate false localization rates (FLRs), deltadot score and F-value were used. Deltadot score reflects how much the first spectral match differs from the second best match in dot product scores and contributes to the total discriminant score F-value [51]; however, deltadot score in the default condition of SpectraST does not consider homologous peptides until the forth match (HOM4 deltadot score). Therefore, the searches were repeated to calculate HOM1 deltadot score by comparing the first and second matches even if they represent the same peptide sequence with only difference in phosphorylation sites. Narrower precursor ion mass tolerance in SpectraST searching may affect the scoring for FLR calculation (S1 Fig); therefore, in this study we decided to use 3 *m/z* tolerance as described above, and 0.05 *m/z* tolerance was subsequently applied to search results prior to the localization. Search results obtained under different simulation conditions were compared (see details in the Result section).

### MaxQuant database search and site localization

The TripleTOF data of K562 and synthetic phosphopeptides were searched against the SwissProt 2010_03 database with MaxQuant [43] version 1.5.5.1. The following search condition was used: carbamidomethylation (C) as a fixed modification, and oxidation (M), phosphorylation (S, T, and Y) and acetylation (protein N-terminus) as variable modifications. Trypsin was specified as an enzyme and two missed cleavage sites were allowed. Precursor and fragment ion mass tolerances were set to 0.05 Da. A 1% FDR cutoff was applied for the K562 phosphopeptides, but not for the synthetic phosphopeptides. PTM score [10] was used for FLR estimation.

## Results and discussion

### Simulated spectral library searching on synthetic phosphopeptide datasets acquired using TripleTOF instrument

**Fig 3:**
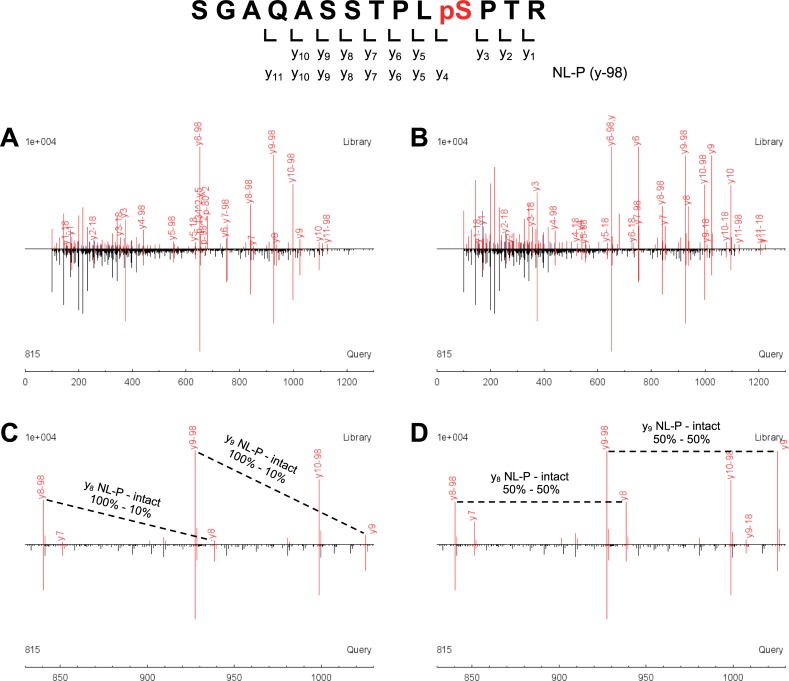
Phosphopeptide simulation on TripleTOF CID spectra. Phosphopeptides were subjected to enzymatic dephosphorylation, TripleTOF CID analysis, and then spectral simulation of single phosphorylations (refer to Fig 2). (A) A representative SpectraST spectral match of a synthetic phosphopeptide (lower spectrum; SGAQASSTPLpSPTR, pS: phosphoserine) with a simulated phosphopeptide (upper spectrum) is shown. Serine-phosphorylated fragment ions (pS intact ions) and their phosphoric acid neutral loss ions (NL-P ions) were predicted from nonphosphorylated ions with 10% and 100% intensities, respectively (refer to Fig 1). (B) The same match is shown, but with the peptide simulated using 50%-50% intensities. (C and D) Fragment ions at *m/z* 830–1030 in the spectral matches A and B are shown, respectively. In the synthetic phosphopeptide spectra, y-series ions matched to those of simulated phosphopeptides are shown in red.

To investigate whether the simulated spectral library approach would be applicable to TripleTOF CID data, we used 62 singly phosphorylated synthetic peptides (62 phosphorylation sites on 24 sequences, refer to S1 Table). The tryptic phosphopeptides, which tended to be differentially localized/identified from the human HeLa cell dataset reported in our previous study [39], were selected for synthetic phosphopeptides to clearly observe localization performance of simulated spectral library searching. For creating a spectral library of simulated phosphopeptides, the synthetic phosphopeptides were enzymatically dephosphorylated and analyzed by LC-MS/MS using a TripleTOF 6600 instrument. The analysis was performed in triplicates without using exclusion time, resulting in acquisition of many spectral replicates for each peptide. X!Tandem database searching was performed against the sequence database (SwissProt 2010_03 human) used for obtaining the HeLa dataset in the previous study [39]. From the dephosphorylated sample data, 3737 peptide spectra were identified with PeptideProphet minimum probability of 0.95, of which nonphosphorylated peptides (3728 spectra, 0.63% FDR) were subjected to spectral simulation of single phosphorylation using SimPhospho [39, 41]. Since the latest version of SimPhospho offers various options on its user interface [41], different simulation conditions can be used. Serine/threonine phosphorylated fragment ions (pS/pT intact ions), their phosphoric acid neutral loss ions (NL-P ions), and tyrosine phosphorylated fragment ions (pY intact ions) were predicted from nonphosphorylated fragment ions (a, b, and y-series) and their ammonia and water neutral loss ions (NL-A and -W) (refer to Fig 1). As the default condition, intensities of pS/pT intact ions, NL-P ions, and pY intact ions relative to nonphosphorylated ions (a, b, y, and NL-AW) were set to 10%, 100%, and 100%, respectively, as used in the original study [39]. The simulated spectra were used to build a spectral library (181 phosphopeptide consensus spectra, 181 decoys) using SpectraST. LC-MS/MS analysis of the synthetic phosphopeptides was performed in triplicates as well, and the acquired TripleTOF CID spectra were subjected to SpectraST searching (version 5 scoring) against the library. Because of the known sequences and data simplicity, no FDR cutoff was applied to the search results. Representative TripleTOF spectral matches shown in Fig 3 demonstrate well-predicted fragment ions in simulated spectra.

**Fig 4:**
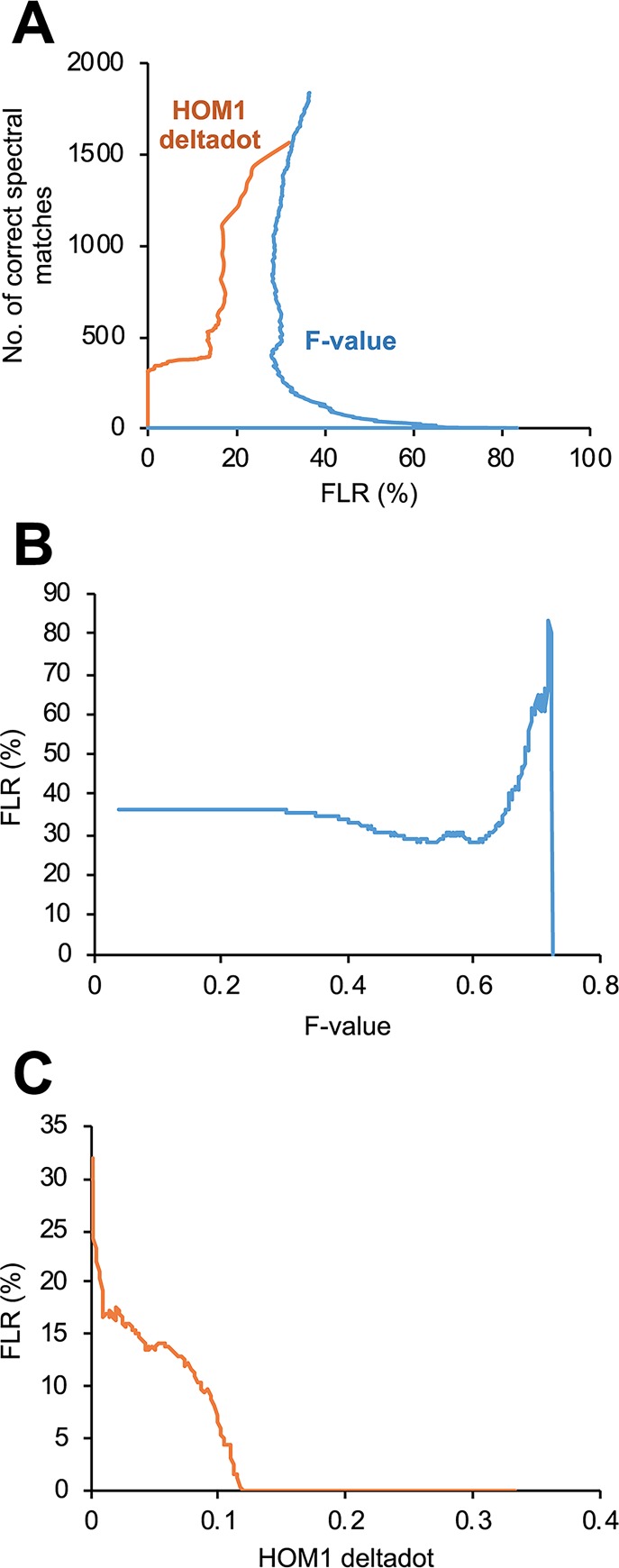
Phosphorylation site localization on synthetic phosphopeptides by simulated spectral library searching. (A) The synthetic phosphopeptides analyzed by TripleTOF CID were searched by SpectraST against the simulated spectral library and the results were sorted by one of the SpectraST scores, F-value or HOM1 deltadot. (B) FLRs as a function of F-value are shown. (C) FLRs as a function of HOM1 deltadot score are shown.

TripleTOF spectral matches of the synthetic phosphopeptides were sorted by the total discriminant score F-value, and then FLRs across F-value were calculated, as described previously for Orbitrap HCD spectral matches [39]. However, only 1 correct spectral match was observed at 1% FLR (Fig 4A), which was due to many false localization matches showing high F-value (Fig 4B). Therefore, the searches were repeated to calculate another discriminant score HOM1 deltadot, of which concept is similar to Mascot delta score [11]. Sorting of the TripleTOF spectral matches by HOM1 deltadot score showed the greater number of correct spectral matches (i.e. true localizations) than the F-value sorting at 0–32% FLRs (Fig 4A). HOM1 deltadot score of 0.116 as a cutoff satisfied 1% FLR for the search result (Fig 4C), with 318 correct spectral matches (Fig 4A).

**Fig 5:**
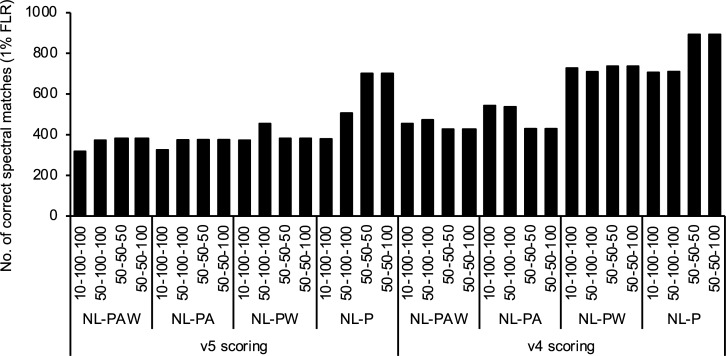
Optimization of simulation conditions for localizing phosphorylation sites on synthetic phosphopeptides. Different simulation conditions were used for creating TripleTOF spectral libraries from the dephosphorylated synthetic peptides. Four different intensities were used for pS/pT intact ions, NL-P ions, and pY intact ions: 10%-100%-100%, 50%-100%-100%, 50%-50%-50%, and 50%-50%-100% (refer to Fig 3 and [39]). Those ions were predicted from nonphosphorylated fragment ions: a, b, y, and their ammonia (A) and water (W) NL ions. Four different NL conditions were used: NL-PAW, NL-PA, NL-PW, and NL-P. Against the 16 libraries, the synthetic phosphopeptides were searched by SpectraST with scoring versions 4 and 5.

To optimize the simulated spectral library search conditions for TripleTOF CID data, the synthetic phosphopeptide spectra were searched using two versions of SpectraST scoring (4 and 5) against 16 spectral libraries created under different simulation conditions. The simulation conditions used were as follows: intensities of pS/pT intact ions, NL-P ions, and pY intact ions were 10%-100%-100%, 50%-100%-100%, 50%-50%-100%, and 50%-50%-50%, and NL conditions were NL-PAW, NL-PA, NL-PW, and NL-P. Total 32 searches were performed, and spectral matches of the synthetic phosphopeptides were sorted by HOM1 deltadot score (see S2 Table for 1% FLR cutoffs). In the number of correct spectral matches at 1% FLR, the searching with version 4 scoring was better than the newest scoring version 5 for all tested simulation conditions (Fig 5). In particular, both 50%-50%-100% and 50%-50%-50% with NL-P showed the highest numbers at 1% FLR, i.e. 893 correct spectral matches (HOM1 deltadot score of 0.017) and 701 correct spectral matches (HOM1 deltadot score of 0.046) with the scoring versions 4 and 5, respectively. This result is consistent with the previous evaluation as 50%-50%-50% was the best condition for the Orbitrap HCD dataset of 20 synthetic phosphopeptides [41]. However, the intensity condition was not enough, and the thorough comparison of the combinations of conditions found that the NL conditions were also critical as 50%-50%-50% with NL-P resulted in 1.8–2.1 times more correct spectral matches than those with NL-PAW used in the previous study [41]. These results suggest that considering NL-A and NL-W ions to be phosphorylated in the simulation may introduce possible simulation errors, and/or somehow interfere with subsequent spectral searching due to unexpected fragment ion matching. The synthetic phosphopeptide results obtained under one of the optimized conditions (50%-50%-100%, NL-P, version 4) are listed in S3 Table.

### Simulated spectral library searching on K562 phosphopeptide datasets acquired using TripleTOF instrument

**Fig 6:**
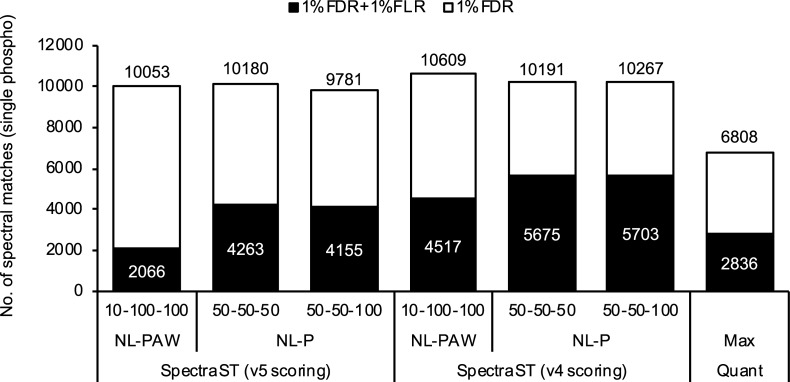
Phosphorylation site localization on K562 phosphopeptides by simulated spectral library searching and MaxQuant searching. From the K562 phosphopeptides analyzed by TripleTOF CID, singly phosphorylated peptides were identified at 1% FDR by SpectraST with the simulated spectral library and by MaxQuant, followed by applying 1% FLR cutoff. Three simulated spectral libraries generated under the default condition (10%-100%-100% intensities, NL-PAW) and the optimized conditions (50%-50%-50% and 50%-50%-100% intensities, NL-P; refer to Fig 5) were used for SpectraST searching with scoring versions 4 and 5.

Simulated spectral library searching for TripleTOF CID data was evaluated on singly phosphorylated peptides obtained from human cell proteins. Phosphopeptides enriched from a K562 tryptic digest and their enzymatically dephosphorylated peptides were analyzed by TripleTOF LC-MS/MS in duplicates using dynamic exclusion. X!Tandem searching of the dephosphorylated sample data identified 13190 human peptide spectra with PeptideProphet minimum probability of 0.95, of which nonphosphorylated peptides (13103 spectra, 0.77% FDR) were subjected to SimPhospho spectral simulation under the default and optimized conditions. Although the K562 proteins were supplemented with a phosphoprotein standard bovine α-casein (S2 Fig), it was not taken into consideration since the number of human peptide spectra obtained was sufficient for the purpose of this study. Against libraries created with the simulated spectra (16752 phosphopeptide consensus spectra for 4182 sequences, 16752 decoys, in each library), SpectraST searching of the phosphopeptide sample data was performed with the scoring versions 4 and 5. At 1% FDR estimated by PeptideProphet, the default condition (10%-100%-100%, NL-PAW) with the version 4 scoring showed the highest number of spectral matches, i.e. 10609 spectral matches (Fig 6). However, after applying the respective 1% FLR cutoffs (HOM1 deltadot scores) obtained from the synthetic phosphopeptide data (S2 Table), one of the optimized conditions (50%-50%-100%, NL-P) with the version 4 scoring showed the highest number (5703 spectral matches), which was 2.8 times higher than that obtained under the default condition with the newer scoring version (2066 spectral matches). As seen on the synthetic phosphopeptide data (Fig 5), the version 4 scoring was better than the version 5 under the three simulation conditions tested on the K562 data (Fig 6). But the newer scoring may have worked less efficiently only with the libraries tested in this study, and further evaluations would be required for their performance comparison, e.g. with larger libraries. A recent sequence database (SwissProt 2018_02 release) was also tested for creating a simulated spectral library but it showed similar results (S2 Fig). The K562 results obtained under the optimized condition (50%-50%-100%, NL-P, version 4) are listed in S4 Table.

To comparatively evaluate the performance of simulated spectral library searching optimized on the TripleTOF CID data, the same data were analyzed using a widely used software MaxQuant [43], where Andromeda engine for database searching [52] and PTM score for phosphorylation site localization [10] were implemented. MaxQuant searching was performed against the same sequence database (SwissProt 2010_03 human). An FLR of 1% on the 62 synthetic phosphopeptides identified with no FDR cutoff required PTM score of 0.977 (S3 Fig). The PTM score obtained on the TripleTOF dataset was somewhat less stringent compared to those previously obtained on the Orbitrap HCD datasets of 20 synthetic human phosphopeptides (0.992) [39] and >100000 synthetic phosphopeptides (0.995) [53]. At 1% FDR and 1% FLR (PTM score ≥0.977) on the K562 singly phosphorylated peptides, MaxQuant searching showed 2836 spectral matches, which were 2.0 times fewer than those obtained by simulated spectral library searching under the optimized condition (Fig 6). The superior identification sensitivity of simulated spectral library searching would largely be due to the limited search space, as spectral libraries contain only peptide spectra confidently identified by database searching. However, the confident localization on identified peptide sequences was achieved by optimization of spectral simulation and search conditions (refer to Figs 4–6).

**Fig 7:**
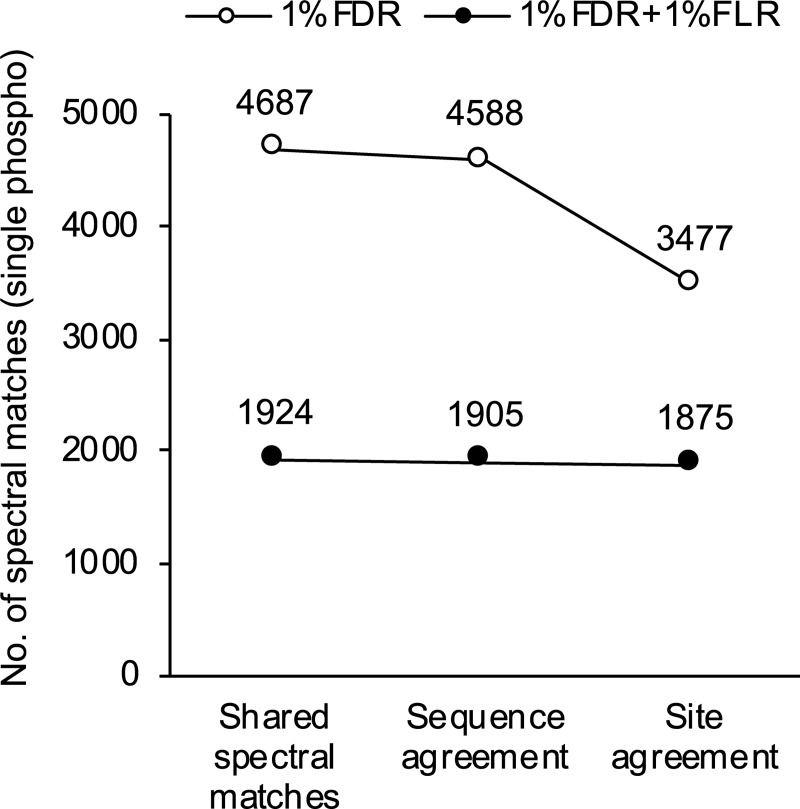
Sequence and site agreements of simulated spectral library searching and MaxQuant searching on K562 phosphopeptides. From the singly phosphorylated K562 peptides analyzed by TripleTOF CID, spectral matches shared by SpectraST searching against the simulated spectral library under the optimized condition (50%-50%-100% intensities, NL-P, versions 4 scoring) and MaxQuant searching were extracted at 1% FDR and 1% FLR (see Fig 6). Out of those, sequences and phosphorylation sites agreed by the two searches were counted.

The K562 results obtained by simulated spectral library searching under the optimized condition and MaxQuant searching shared 4687 spectral matches at 1% FDR (Fig 7). Among those, the two searches agreed on 4588 spectral matches for their peptide sequences (97.9%). Assuming that all the agreeing search results are correct identifications, the search disagreement (2.1%) is consistent with the expectation that each search may contain 1% false sequence identifications. The two searches agreed only on 3477 spectral matches for their phosphorylation sites on the agreed sequences (75.8%). However, at 1% FDR and 1% FLR, the site agreement was clearly improved to 98.5% (i.e. 1.5% disagreement) as the two searches agreed on 1905 and 1875 spectral matches for their peptide sequences and phosphorylation sites, respectively. These observations support the validity of the FLR cutoff scores obtained on the synthetic phosphopeptides, and also the quality of the results obtained by simulated spectral library searching.

## Conclusions

In this study, simulated spectral library searching previously proposed for Orbitrap HCD data was evaluated on TripleTOF CID data. SimPhospho enabled the simulation of single phosphorylations on TripleTOF CID spectra, and facilitated the use of various simulation conditions. After optimization for the TripleTOF CID data, simulated spectral library searching with SpectraST achieved highly confident and sensitive localization of phosphorylation sites. However, development of simulation algorithm for multiply phosphorylated peptides, evaluation of compatibility with alternative spectral library software, and further optimization for both TripleTOF CID and Orbitrap HCD remain to be considered. In principle, simulation of multiple phosphorylations might be performed by multiplying the mass shifts used for single phosphorylation, but careful evaluation and optimization would be needed with some model data. Although creation of simulated spectral libraries requires additional steps, such as enzymatic dephosphorylation, CID measurement, and finally spectral simulation in each experiment, the resulting libraries will cover all the possible phosphorylation sites on stored high-confidence peptide sequences and can be used repeatedly in a large-scale experiment, as demonstrated in our previous study [23]. Because of limited sizes of spectral libraries, simulated spectral library searching can be used to supplement database search results as mentioned above [23]. Alternatively, phosphopeptides may be simulated based on publicly available large spectral libraries such as PeptideAtlas [54]. Once de novo simulation of beam-type CID spectra for all the theoretical tryptic peptides would be achieved with well-predicted ion intensities, it may increase the impact of the phosphorylation simulation on sensitive and confident localization of phosphorylation sites.

## Supporting information

S1 Table List of synthetic phosphopeptides. (XLSX)

S2 Table HOM1 deltadot scores required for 1% FLR on synthetic phosphopeptides under different simulation conditions. (XLSX)

S3 Table Synthetic phosphopeptides identified and localized by simulated spectral library searching under the optimized condition (50%-50%-100%, NL-P, version 4). (XLSX)

S4 Table K562 phosphopeptides identified at 1% FDR and localized by simulated spectral library searching under the optimized condition (50%-50%-100%, NL-P, version 4). (XLSX)

S1 Fig Simulated spectral library searching of synthetic phosphopeptides under different mass tolerance conditions. Different precursor ion mass tolerance conditions (0.05 *m/z*, 0.1 *m/z*, 1 *m/z*, 3 *m/z*, and 5 *m/z*) were tested for simulated spectral library searching. For this evaluation, the previously reported Orbitrap HCD datasets were used (ref. 39: Suni *et al*., 2015). A simulated spectral library was created from HeLa dephosphorylated peptides under the default condition (10%-100%-100% intensities, NL-PAW), and used for SpectraST searching (version 4 scoring) of 20 synthetic phosphopeptides. The search results for the different mass tolerances were further filtered by the tolerance of 0.05 *m/z*. Those results were sorted by F-value for FLR calculation. The searching with 3 *m/z* tolerance showed more spectral matches at 1% FLR than those with the narrower tolerances, but the post-search filter by 0.05 *m/z* did not reduce the matches significantly. These results suggest that the mass tolerance condition in SpectraST searching may affect scoring used for FLR caluculation. Therefore, we decided to use 3 *m/z* tolerance in combination with 0.05 *m/z* post-search filter for the localization in this study.
(PDF)

S2 Fig Searching of K562 and casein phosphopeptides against a simulated spectral library created with a recent sequence database. A SwissProt database (2018_02 release, *Homo sapiens*) supplemented with common contaminants (forward-reverse, total 40762 sequences) was tested for creating a simulated spectral library (50%-50%-100% intensities, NL-P). SpectraST searching (version 4 scoring) against the library showed 3266 spectral matches for casein phosphopeptides at 1% FDR, in addition to 10604 spectral matches for human phosphopeptides. Refer to Fig 6 for the search without casein phosphopeptides.
(PDF)

S3 Fig MaxQuant searching of synthetic phosphopeptides for calculating FLRs across PTM score. The TripleTOF 6600 dataset (9 wiff files) of the 62 singly phosphorylated synthetic peptides (24 human peptide sequences) was searched with MaxQuant. The searches resulted in 1058 spectral matches for the synthetic peptides, and 1% FLR required PTM score of 0.977.
(PDF)
