# Supplementary material for: Optimization of TripleTOF spectral simulation and library searching for confident localization of phosphorylation sites
Source: PLoS One. 2019 Dec 2;14(12):e0225885. doi: 10.1371/journal.pone.0225885 (PMC6886777; doi:10.1371/journal.pone.0225885)
Supplement: S1 Fig — Different precursor ion mass tolerance conditions (0.05 m/z, 0.1 m/z, 1 m/z, 3 m/z, and 5 m/z) were tested for simulated spectral library searching. For this evaluation, the previously reported Orbitrap HCD datasets were used (ref. 39: Suni et al., 2015). A simulated spectral library was created from HeLa dephosphorylated peptides under the default condition (10%-100%-100% intensities, NL-PAW), and used for SpectraST searching (version 4 scoring) of 20 synthetic phosphopeptides. The search results for the different mass tolerances were further filtered by the tolerance of 0.05 m/z. Those results were sorted by F-value for FLR calculation. The searching with 3 m/z tolerance showed more spectral matches at 1% FLR than those with the narrower tolerances, but the post-search filter by 0.05 m/z did not reduce the matches significantly. These results suggest that the mass tolerance condition in SpectraST searching may affect scoring used for FLR caluculation. Therefore, we decided to use 3 m/z tolerance in combination with 0.05 m/z post-search filter for the localization in this study. (PDF) [file pone.0225885.s005.pdf]

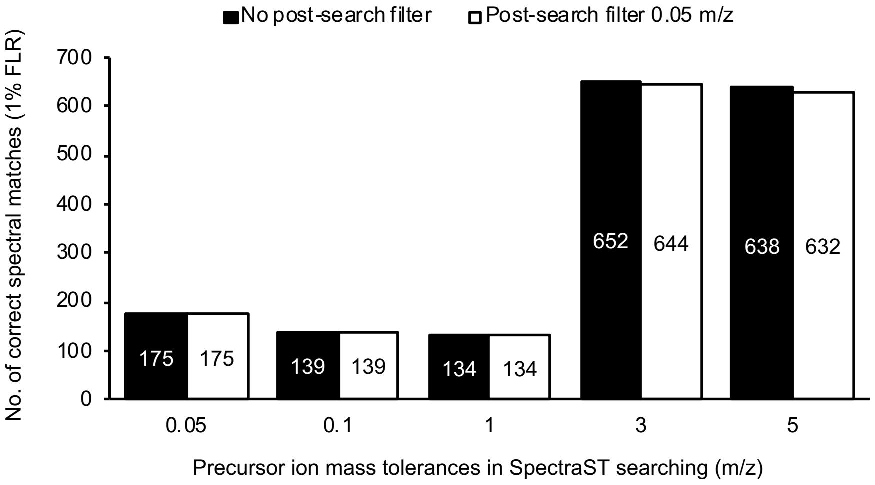

S1 Fig. Simulated spectral library searching of synthetic phosphopeptides under different mass tolerance conditions.

Different precursor ion mass tolerance conditions (0.05 *m/z*, 0.1 *m/z*, 1 *m/z*, 3 *m/z*, and 5 *m/z*) were tested for simulated spectral library searching. For this evaluation, the previously reported Orbitrap HCD datasets were used (ref. 39: Suni *et al.*, 2015). A simulated spectral library was created from HeLa dephosphorylated peptides under the default condition (10%-100%-100% intensities, NL-PAW), and used for SpectraST searching (version 4 scoring) of 20 synthetic phosphopeptides. The search results for the different mass tolerances were further filtered by the tolerance of 0.05 *m/z*. Those results were sorted by F-value for FLR calculation. The searching with 3 *m/z* tolerance showed more spectral matches at 1% FLR than those with the narrower tolerances, but the post-search filter by 0.05 *m/z* did not reduce the matches significantly. These results suggest that the mass tolerance condition in SpectraST searching may affect scoring used for FLR caluculation. Therefore, we decided to use 3 *m/z* tolerance in combination with 0.05 *m/z* post-search filter for the localization in this study.
