# Supplementary material for: Optimization of TripleTOF spectral simulation and library searching for confident localization of phosphorylation sites
Source: PLoS One. 2019 Dec 2;14(12):e0225885. doi: 10.1371/journal.pone.0225885 (PMC6886777; doi:10.1371/journal.pone.0225885)
Supplement: S2 Fig — A SwissProt database (2018_02 release, Homo sapiens) supplemented with common contaminants (forward-reverse, total 40762 sequences) was tested for creating a simulated spectral library (50%-50%-100% intensities, NL-P). SpectraST searching (version 4 scoring) against the library showed 3266 spectral matches for casein phosphopeptides at 1% FDR, in addition to 10604 spectral matches for human phosphopeptides. Refer to Fig 6 for the search without casein phosphopeptides. (PDF) [file pone.0225885.s006.pdf]

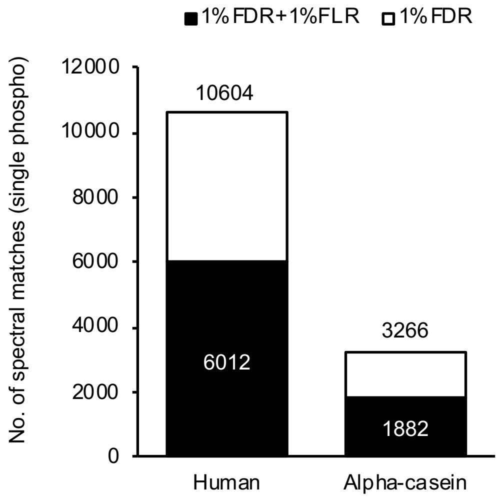

S2 Fig. Searching of K562 and casein phosphopeptides against a simulated spectral library created with a recent sequence database.

A SwissProt database (2018_02 release, *Homo sapiens*) supplemented with common contaminants (forward-reverse, total 40762 sequences) was tested for creating a simulated spectral library (50%-50%-100% intensities, NL-P). SpectraST searching (version 4 scoring) against the library showed 3266 spectral matches for casein phosphopeptides at 1% FDR, in addition to 10604 spectral matches for human phosphopeptides. Refer to Fig 6 for the search without casein phosphopeptides.
