# Supplementary material for: Optimization of TripleTOF spectral simulation and library searching for confident localization of phosphorylation sites
Source: PLoS One. 2019 Dec 2;14(12):e0225885. doi: 10.1371/journal.pone.0225885 (PMC6886777; doi:10.1371/journal.pone.0225885)
Supplement: S3 Fig — The TripleTOF 6600 dataset (9 wiff files) of the 62 singly phosphorylated synthetic peptides (24 human peptide sequences) was searched with MaxQuant. The searches resulted in 1058 spectral matches for the synthetic peptides, and 1% FLR required PTM score of 0.977. (PDF) [file pone.0225885.s007.pdf]

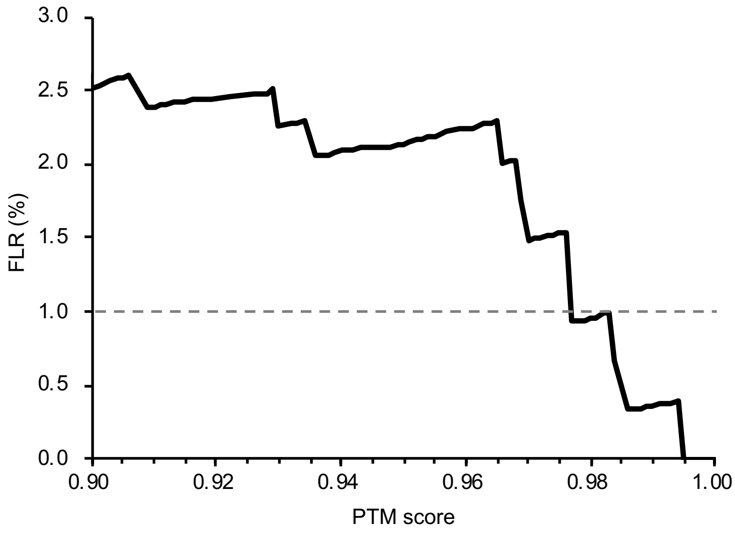

S3 Fig. MaxQuant searching of synthetic phosphopeptides for calculating FLRs across PTM score.

The TripleTOF 6600 dataset (9 wiff files) of the 62 singly phosphorylated synthetic peptides (24 human peptide sequences) was searched with MaxQuant. The searches resulted in 1058 spectral matches for the synthetic peptides, and 1% FLR required PTM score of 0.977.
